# Burden and Correlates of Falls among Rural Elders of South India: Mobility and Independent Living in Elders Study

**DOI:** 10.1155/2017/1290936

**Published:** 2017-06-13

**Authors:** Pawan Kumar Sharma, Clareann H. Bunker, Tushar Singh, Enakshi Ganguly, P. Sudhakar Reddy, Anne B. Newman, Jane A. Cauley

**Affiliations:** ^1^SHARE India, MediCiti Institute of Medical Sciences, Telangana, India; ^2^Department of Epidemiology, University of Pittsburgh, Graduate School of Public Health, Pittsburgh, PA, USA

## Abstract

**Aim:**

Falls are an important contributor to loss of function, morbidity, and mortality in elders. Little is known about falls in Indian populations. The objective of this cross-sectional report was to identify the prevalence and correlates of falls in a cohort of 562 rural southern Indian men and women.

**Methods:**

Risk factors included demographics, anthropometrics, self-reported health, medical history, physical function, vision, depression, and lifestyle. Odds ratios were calculated using logistic regression.

**Results:**

71 (13%) subjects reported at least 1 fall in the past year. Prevalence was higher among women (17%) than men (8%), *P* = 0.003. Sex and age showed significant interaction (*P* = 0.04) whereby falls prevalence increased with age among women but decreased among men. Correlates of falls among men included a history of osteoarthritis (OA) (odds ratio (OR): 6.91; 95% CI: 1.4–33.1), depression (OR:9.6; 3.1–30.1), and greater height (OR per 1 standard deviation increase: 2.33; 1.1–5.1). Among women, poor physical performance (OR: 3.33; 1.13–9.86) and history of cardiovascular disease (CVD) (OR: 2.42; 1.01–5.80) were independently associated with falls.

**Implications:**

Prevalence of falls in elderly South Indians was lower than published reports from western countries and likely reflects low exposure to fall risks. Patterns with age differed in men and women and may reflect sex differences in the accuracy of age recall. Presence of comorbidities specifically OA, CVD, and depression was independent correlate of falling.

## 1. Introduction

Falls are an important health problem among elderly populations worldwide, with reported global incidences ranging from 224 to 809 per 1000 person-years [1–6]. They are a major cause of injuries and resulting disabilities [7], impairing functional activities of daily living [7–10], and quality of life. Information on falls epidemiology among older adults from developing countries is limited [1, 11, 12]. Studying falls in the Indian population is important in view of the current demographic transition in the country that is leading to a rapid increase in the elderly population [13].

A recent cross-sectional study among older adult men and women aged 60 and above in northern India reported that 52% of subjects fell in the past year [14], suggesting a high burden. The prevalence of falls in hospitalized or institutionalized Indian women was also very high, 45% to 64% [15]. However, to our knowledge no population based study has described correlates of falls among Indian elders. Moreover, there is lack of data from rural populations, where the majority of Indian elders reside.

The present paper was designed to identify the prevalence of falls over the past 12 months and to describe correlates of falls among a cohort of rural older South Indians. We hypothesized that correlates of falls would be similar to risk factors identified in western populations.

## 2. Methods

The “Mobility and Independent Living in Elders Study” (MILES), a cohort study with the primary objective to study risk factors for mobility disability among elders aged ≥ 60 years, was implemented in a South central part of India, Telangana state in 2012. We analyzed cross-sectional data collected between February 2012 and November 2012 from the population aged 60 years and over who participated in MILES. We identified 863 elders eligible for the study. Of these 562 (68.0%) men and women (50% women; 50% men) agreed to participate. One participant was dropped from analysis because of missing data. The final number of participants included for the present analysis was 280 men and 281 women (total = 561). The MILES design and recruitment paper describing the cohort has been published [16]. Briefly, individuals were randomly selected from ten villages using the Population Proportion to Size (PPS) method out of a total of 31 villages in sampling frame in Medchal region of Telangana state (previously Andhra Pradesh), India. Each participant completed an interview and clinic examination. The study was ethically approved by the Institutional Review Boards of SHARE India and the University of Pittsburgh. The international collaboration was approved by the Indian Council of Medical Research (ICMR). All subjects were required to provide written informed consent using a locally translated consent form.

Inclusion criteria were Indian, age ≥ 60 years old, living at home in Medchal mandal, and able to walk independently or with a walking aid.

Ascertainment of falls was similar to methods used in population based studies in the US (SOF, MrOS, and Health ABC) [17–19]. A person was defined as a faller if s/he answered affirmatively to the following questions: “Have you fallen in the past 12 months?” and “If so, how many times?” Recurrent fallers were characterized as reporting two or more falls in the previous year.

*Demographic and Health Information*. Data was collected by trained and certified clinical staff using questionnaires adapted from WHO Study of Global AGEing and Adult Health (SAGE) [20], Health Aging and Body Composition study (Health ABC) [21], and The Lifestyle Interventions and Independence for Elders (LIFE) Pilot Study [22]. We collected information on age, sex, marital status, education, self-reported general health, medical history, and physical function. Hearing was defined as poor in a person who self-reported poor hearing or complete deafness. Presence of depressive symptoms was assessed using the Geriatric Depression Scale (15 item) (GDS) [23]; respondents with a score of >5 were considered to have depression. Vision was described as self- reported poor or very poor vision.

Activities of daily living (ADLs) [24] included walking across a room, dressing, bathing, eating, getting in and out of bed, and using the toilet. If participants needed help or were unable to perform one or more of these six ADLs, they were considered functionally impaired. A physically active participant was defined as one who answered affirmatively to the question “do you regularly exercise such as jogging, dance, or perform rigorous physical activity at least for 10 minutes three times weekly for the past year?” Lower extremity physical limitation was considered present if the participant expressed that s/he had difficulty standing up from the floor, climbing stairs, stooping, kneeling, crouching, standing for long periods, or walking for long or short distances.

*Anthropometric Measurements*. Height was measured using Seca 214 Stadiometer (Seca, Hanover, MD). Weight (using Seca 813 Digital Scale (Seca, Hanover, MD)) was measured with very light clothing and the body mass index was calculated (kg/m^2^). Systolic and diastolic blood pressure was recorded in the left arm, sitting position, using a digital monitor, Omron Hem-705 (Omron Healthcare, Inc., Lake Forest, IL).

*Tests of Mobility Disability and Muscle Strength*. The Short Physical Performance Battery (SPPB) includes a 4-meter walk, repeated chair stands, and three hierarchical standing balance tests [25]. Each of the three performance measures was assigned a categorical score ranging from 0 to 4, with 4 indicating the highest level of performance and 0 the inability to complete the test. A summary score ranging from 0 (worst performers) to 12 (best performers) was calculated by adding the gait speed, chair stands, and balance scores. Participant with scores 0–9 were defined as having poor SPPB.

Participants walked 400 meters on a 20-meter course (10 laps) in an open corridor. Mobility disability was defined objectively as the inability to attempt or complete the 400-meter self-paced walk within 15 min without sitting or the use of an assistive device (including a cane) or the help of another person [22].

Grip strength was measured in both hands using an isometric dynamometer (Jamar, Lafayette Instrument, Lafayette, IN). An average of the two readings of dominant hand was used in the analysis.

### 2.1. Statistical Analysis

Data was analyzed using SPSS, version 21 software (SPSS Inc., Chicago, IL, USA). Comparison of characteristics of fallers and nonfallers was done using the chi-square test for categorical variables and the *t*-test for continuous variables. We created three models: men, women, and the total study population, adjusted for sex. The variables that emerged statistically significant (*P* value < 0.10) in the univariate analysis were selected for multivariable models. Specifically, we examined age, marital status (single versus living with spouse), osteoarthritis (OA), cardiovascular disease (CVD), stroke, depression, mobility difficulty, diastolic BP, SPPB (≤9), and BMI. We used SPPB in the models as a measure of physical performance since it is a composite measure of three important parameters of mobility. We performed backward elimination logistic regression testing the deletion of each variable using a chosen model comparison criterion, deleting the variable that improves the model the most and repeating this process until no further improvement was possible, to get final set of the independent predictor variables. Variables with *P* value of <0.15 were retained in the final model. The same sets of variables were used for each model. Results of the logistic regression are presented as odds ratios (OR) with 95% confidence intervals (95% CI). For continuous variables, we expressed the OR per 1 standard deviation (SD) increase.

## 3. Results

### 3.1. Study Participants

The total sample consisted of 561 participants, mean age 67.5 ± 6.4 years, Table 1.

### 3.2. Reported Falls in the Last 12 Months

A total of 71 (13%) participants reported a fall in last 12 months. The prevalence of falls was higher in women (17%) than men (8%) (*P* = 0.003). Of the 71 fallers, 44 (62%) reported a single fall, and 27 (38%) reported recurrent falls. Recurrent fallers fell on average 2.5 times.

### 3.3. Correlates of Falls

**Figure 1:**
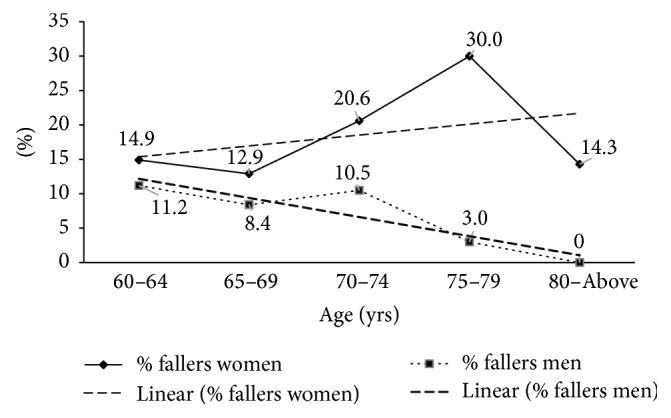
Proportions of men and women who reported falling across age groups.

The mean age of fallers and nonfallers was similar (Table 1) but the proportion who fell in the past year increased across age groups among women, but decreased with age among men, *p* interaction (age × sex) = 0.04, Figure 1.

Upon stratification by sex, mean height, weight, and BMI were all significantly higher in fallers compared to nonfallers (*P* < 0.05) among men but not among women. Self-reported OA (men only) (*P* < 0.006), cardiovascular disease (CVD) (men and women) (*P* < 0.001), and depression (men and women) (*P* < 0.001) were higher in fallers compared to nonfallers. Physical function measures tended to be lower in women who fell compared to women who did not fall. Difficulty in ADLs was higher in women who fell compared to nonfallers (*P* < 0.008). Poor hearing and a history of diabetes, stroke, hypertension, fractures, and measured blood pressure did not differ by fall status. There was also no difference in the proportion who reported difficulty with mobility or no physical activity but >90% of both fallers and nonfallers reported difficulty and no exercise. The only difference in the combined sample of men and women compared to sex specific comparisons was that fallers were more likely to report poor vision.

### 3.4. Multivariate Models of Falls (Yes/No)

#### 3.4.1. Men

Self-report of OA, depression, and greater height were independently associated with a higher risk of falls in men, Table 2. The OR of falling per 1 SD increase in BMI approached statistical significance.

#### 3.4.2. Women

Poor physical function and a history of CVD were independently associated with falls in older women, Table 3. Women who fell were more likely to be depressed but in the final multivariable model this did not reach statistical significance.

#### 3.4.3. Total Population

In the combined sample of men and women, there was no independent effect of sex on fall risk (OR = 1.28; 95% CI, 0.46, 3.56). In models adjusting for sex, fallers were more likely to be depressed and have a history of CVD. A one standard deviation (4.2 kg/m^2^) increase in BMI was associated with a 30% increased likelihood of falling, Table 4.

## 4. Discussion

In this cohort of 561 elders from the rural population in India, 13% of the participants reported falling at least once in the past 12 months. This prevalence is considerably lower than reported in western countries (28–33%) [3, 4, 6–8] and most studies in Japan (12.3–26.9%) [26] and in Hong Kong (20%) [27] and much lower than the 64% prevalence reported among long term care institutionalized persons in India and the 45% prevalence among community living women of urban area of Kerala (Southernmost province of India) [15]. The greater percentage of fallers among women in Kerala may reflect the characteristics of these women: higher mean age (72.6 years); greater physical activity (46% were physically active); and higher prevalence of comorbidity (89% suffered from chronic illnesses, and 73% were on regular medication). The low prevalence of falls in MILES participants may reflect low exposure to risk given the common practice for the rural elderly in India to sit on the floor or ground and negotiate few steps and their lack of exercise.

There was no difference in the mean age between fallers and nonfallers. Nevertheless, we did find the expected increase in the prevalence of falling with age among women but not men and a significant interaction between age and sex. The lack of an association between falling and age in men was surprising given the established association between falls and increasing age [9]. This may reflect a cultural difference, whereby most elderly individuals especially men in rural India are not aware of their dates of birth and may not have a reliable estimate of their current age. We relied on the age in the REACH database (Rural Effective Affordable Health Care), a community census which may be inaccurate [28]. In addition the patterns of activity and exposure to fall risk with age may differ in men and women.

The percentage of fallers was higher among women than men but, in the multivariable analysis, this sex difference was not significant. This finding conflicts with data from Western and Japanese studies whereby fall rates are higher in women [9, 26, 29]. Our lack of significance may reflect our low power to see a sex difference.

Our results confirm findings of many studies that there is an association between falling and depression [8, 30–32]. In our study, depression was more common among women than men and among fallers than nonfallers. Depression was a strong independent risk factor for falling among men (almost 10-fold) as well as for total study population (3-fold); the association was borderline in women. Depression contributes to physical decline [33] and may therefore increase the risk of falling. However, we adjusted for SPPB and ADL impairment and depression remained significant perhaps because depression itself may be a composite indicator of the multiple cognitive and physical deficits that contribute to falls [34]. The association with depression may also reflect use of antidepressant medications which have been linked to falls [31]. However, no one in MILES reported use of antidepressants.

Among women and the total population, history of CVD was associated with a 2–2.5-fold increased risk of falling. Others have also found an association of falling with CVD [35]. CVD may contribute to falls consequent to suffering chest pain, transient ischemic attacks, or stroke. CVD may also result in decreased muscle perfusion due to endothelial dysfunction and decreased arterial compliance, thereby reducing muscle strength [36] leading to a fall. Subjects with CVD are often prescribed medication that may cause orthostatic hypotension [37]. These effects may be exaggerated in older women compared to men, thus causing women to fall more often than men

OA was an independent risk factor for falls with over a 7-fold increased risk of falling in men but not women. OA is one of the most important contributors to disability in older populations [38] and the overall prevalence of OA was high in all subjects. Others have also reported a higher proportion of falls among elderly men with OA [39]. We have no information on the specific site of OA and it is possible that more men had knee or hip OA that contributed to the increased risk of falls. Knee OA has been associated with poor balance control which could precipitate falls [40].

Poor physical function was associated with a 3-fold increased risk of falling in women but not men. The prevalence of poor physical performance was much higher in women than men. Over 90% of women who fell compared with 50% of men who fell had a low SPPB score. This higher prevalence among women may have contributed to our observation that poor SPPB was related to falls in women but not men. Women are more likely to have a frailer phenotype compared to men and have more neurological symptoms that present as pain, tingling or weakness in the lower limbs, and weight bearing joints causing them to lose their balance and fall more often. However, other studies have shown that physical function was an important predictor of falling among older men [10].

A one standard deviation increase in BMI was associated with a 30% increased risk for falling in the total study population. This association was not observed in gender specific models and may reflect greater power in the combined sample. Overall, the BMI in this population was very low suggesting a lean group of men and women. It is possible that we did not see stronger relationships with BMI because of our narrow distribution of BMI. For example, only 5% of subjects had a BMI > 30 kg/m^2^ which has been associated with an increased risk of falls [41]. Taller men were twice as likely to fall. Previous studies have not consistently reported height as a risk factor for falls. Indeed shorter height was associated with more falls in older women enrolled in the Study of Osteoporotic Fractures [42].

There are a number of strengths to our study. To our knowledge this is the largest population based study of falls in rural elders in India. Our study staff was certified in data collection/management procedures by the University of Pittsburgh Center for Healthy Aging; and Johns Hopkins University. Our participants were selected randomly for adequate community representation. However, there are a number of limitations. First, we used the age recorded from a community census which may be inaccurate. Rural Indian elderly seldom remember their year of birth due to the absence of a formal birth registration system. There may be recall bias for falls contributing to our low prevalence of falls. Our low prevalence of falls may also have limited our power to see associations. The overall prevalence of disability was high in both fallers and nonfallers limiting our ability to see differences by fall status. Finally, our findings are cross-sectional and need to be confirmed in prospective studies.

Although the percentage of fallers was relatively low, our findings have important public health implications for India. The Indian elderly population (≥65) is expected to expand rapidly to about 13% in 2050. Risk of falls may also increase because of the increasing prevalence of chronic disease such as CVD [43].

## 5. Conclusion

The prevalence of falls in elderly South Indians was lower than published reports from western countries and likely reflects low exposure to fall risks. Prevalence of falls increased with age in women but declined with age in men which may reflect sex differences in the accuracy of age recall or differences in exposures to fall risk. Presence of comorbidities specifically OA, CVD, and depression was independent correlate of falling.

## Figures and Tables

**Table 1:** Characteristics of men and women by fall status.

Risk factors	Women (*N* = 281)		*P* value	Men (*N* = 280)		*P* value	Total (*N* = 561)		*P* value
	Percent or mean ± SD			Percent or mean ± SD			Percent or mean + SD		
	Nonfallers (*n* = 234)	Fallers (*n* = 47)		Nonfallers (*n* = 256)	Fallers (*n* = 24)		Nonfallers (*n* = 490)	Fallers (*n* = 71)	
*Demographic *									
Age (years) (mean)^*∗*^	67.03 ± 6.1	68.15 ± 6.6	0.26	68.18 ± 6.7	65.75 ± 4.3	0.08	67.63 ± 6.49	67.34 ± 6.05	0.16
Height (cm) (mean)^*∗*^	147 ± 5.9	146 ± 5.7	0.59	160 ± 5.7	163 ± 5.0	**0.03**	154.24 ± 8.85	152.22 ± 9.53	0.08
Weight (kg) (mean)^*∗*^	50.12 ± 11.8	51.66 ± 15.2	0.44	55.09 ± 11.5	61.13 ± 10.4	**0.01**	52.72 ± 11.98	54.85 ± 14.50	0.72
BMI (kg/m^2^) (mean)^*∗*^	22.9 ± 4.69	23.8 ± 5.99	0.30	21.2 ± 3.9	22.9 ± 3.8	**0.04**	22.1 ± 4.38	23.5 ± 5.35	**0.01**
Marital status (single) (%)	59.4	70.2	0.1	10.5	0	**0.078**	33.9	46.5	**0.02**
No education (%)	91.0	93.6	0.40	54.3	37.5	0.08	71.8	74.6	0.36

*Medical history *									
Poor vision (%)	88.0	93.6	0.19	77.3	87.5	0.18	82.4	91.5	**0.03**
Poor hearing (%)	9.0	12.8	0.28	0.4	0	0.91	4.5	8.5	0.12
Osteoarthritis (%)	73.5	80.9	0.19	66.8	91.7	**0.007**	70.0	84.5	**0.006**
Stroke (%)	6.4	10.6	0.22	8.2	16.7	0.15	7.3	12.7	0.09
Hypertension (%)	52.56	57.44	0.32	47.27	54.16	0.33	49.8	56.3	0.18
Cardiovascular disease (%)	65.4	85.1	**0.005**	47.3	70.8	**0.02**	55.9	80.3	**<0.001**
Diabetes (%)	15.38	14.89	0.56	10.93	25	0.054	13.1	18.3	0.15
Fractures (%)	5.6	6.4	0.5	5.1	4.2	0.65	5.3	5.6	0.56
Difficulty in mobility (%) (walking, standing, stooping, keeling, crouching, climbing)	97.4	100	0.33	91.4	87.5	0.36	94.3	95.8	0.43
Use walking aid (%)	18.4	25.5	0.17	27.7	29.2	0.52	23.3	26.8	0.30
Low physical activity (no exercise/no sports) (%)	92.7	95.7	0.35	95.3	91.7	0.34	94.1	94.4	0.59

*Objective measurements*									
Depression: >5 (GDS-15 point scale) (%)	35.5	57.4	**0.004**	8.6	33.3	**0.002**	21.4	49.3	**<0.001**
Grip strength - kg (mean)^*∗*^	12.9 ± 4.7	10.8 ± 4.4	**0.013**	19.7 ± 5.8	21.2 ± 6.6	0.29	16.78 ± 6.38	15.06 ± 7.48	0.098
Short Physical Performance Battery (SPPB) (mean)^*∗*^	7.49 ± 2.7	5.57 ± 2.9	0.000	8.7 + 2.8	8.5 + 3.2	0.82	8.13 ± 2.87	6.60 ± 3.32	**<0.001**
Poor Short Physical Performance Battery (score ≤ 9) (%)	72.2	91.3	**0.003**	53.5	50	0.45	62.4	77.1	**0.01**
Balance (≤3)	31.2	58.7	**<0.001**	18.0	16.7	0.56	24.3	44.3	**0.001**
Gait speed (≤3)	90.6	95.7	0.20	71.5	58.3	0.13	80.6	82.9	0.39
Chair stand test (≤3)	87.6	95.7	0.08	72.7	75.0	0.51	79.8	88.6	**0.05**
400 Meters walk (% who attempted but could not complete + did not attempt)	39.7	63.8	**0.002**	27.0	37.5	0.19	33.1	54.9	**<0.001**
Systolic Blood pressure (mmHg) (mean)^*∗*^	131 ± 22.4	133 ± 23.5	0.48	136 ± 23.4	136 ± 32.0	0.96	133.75 ± 23.13	134.61 ± 26.56	0.77
Diastolic blood pressure (mmHg) (mean)^*∗*^	72 ± 11.25	74 ± 11.12	0.18	72 ± 11.4	75 ± 17.9	0.27	72.37 ± 11.35	74.82 ± 13.70	0.09
Activities of Daily Living (ADL) (% with difficulty)	69.7	87.2	**0.008**	72.3	75.0	0.49	71.0	83.1	**0.02**

*χ* ^2^ statistics, ^*∗*^*t*  test statistics.

**Table 2:** Logistic regression predicting odds of having fallen in the past year by risk factors among Men (Model 1).

Risk factors	Men	
	OR	95% CI
Osteoarthritis^*∗∗∗*^	6.91	1.44, 33.1
Depression^*∗∗∗*^	9.61	3.07, 30.07
Height (per 1 SD increase)^*∗∗*^	2.33	1.05, 5.13

Backward stepwise logistic regression: ^*∗∗*^*P* < 0.05; ^*∗∗∗*^*P* < 0.01; variable(s) in the model: age (per 1 SD), arthritis, depression, diastolic BP (per 1 SD), SPPB, BMI (per 1 SD), cardiovascular disease, stroke, vision, height (per 1 SD), ADL, diabetes, and education.

**Table 3:** Logistic regression predicting odds of having fallen in the past year by risk factors among Women.

Risk factors	Women	
	OR	95% CI
Depression	1.71	0.87, 3.35
Poor physical performance (SPPB ≤ 9)^*∗∗*^	3.33	1.13, 9.86
Cardiovascular disease^*∗∗*^	2.42	1.01, 5.80

Backward stepwise logistic regression: ^*∗∗*^*P* < 0.05; variable(s) in the model: age (per 1 SD), arthritis, depression, diastolic BP (per 1 SD), SPPB, BMI (per 1 SD), cardiovascular disease, stroke, vision, height (per 1 SD), ADL, diabetes, education, and marital status.

**Table 4:** Logistic regression predicting odds of having fallen in the past year by risk factors in study population adjusted with sex.

Risk factors	Total study population	
	OR	95% CI
Depression^*∗∗∗*^	2.97	1.73, 5.11
Body mass index (per 1 SD increase)^*∗∗*^	1.31	1.03, 1.66
Cardiovascular disease^*∗∗*^	2.16	1.13, 4.12

Backward stepwise logistic regression: ^*∗∗*^*P* < 0.05; ^*∗∗∗*^*P* < 0.01; variable(s) in the model: age (per 1 SD), gender, arthritis, depression, diastolic BP (per 1 SD), SPPB, BMI (per 1 SD), cardiovascular disease, stroke, vision, height (per 1 SD), ADL, diabetes, education, and marital status.
